# Role of nonoperative treatment in managing degenerative tears of the medial meniscus posterior root

**DOI:** 10.1007/s10195-013-0234-2

**Published:** 2013-03-27

**Authors:** Devdatta Suhas Neogi, Ashok Kumar, Laxman Rijal, Chandra Shekhar Yadav, Ashish Jaiman, Hira Lal Nag

**Affiliations:** 1 Department of Orthopaedics, All India Institute of Medical Sciences, Ansari Nagar, New Delhi, 110029 India; 2 Department of Orthopaedics, Goa Medical College and Hospital, Bambolim, Goa India; 3 Dubai Bone and Joint Centre, Dubai Healthcare City, Dubai, UAE; 4 Department of Orthopaedics, Civil Lines Hospital, Kathmandu, Nepal; 5 Department of Orthopaedics, Vardhaman Mahavir Medical College and Safdurjung Hospital, New Delhi, India

**Keywords:** Medial meniscus, Meniscal root, Knee osteoarthritis, Exercise

## Abstract

**Background:**

Tears of the medial meniscus posterior root can lead to progressive arthritis, and its management has no consensus. The aim of our study was to evaluate the effect of supervised exercise therapy on patients with medial meniscus posterior root tears.

**Materials and methods:**

Between January 2005 and May 2007, 37 patients with this tear verified by magnetic resonance imaging (MRI) and osteoarthritis grade 1–2 by radiographic examination were treated by a short course of analgesics daily for up to 6 weeks and then as required during follow-up, as well as a 12-week supervised exercise program followed by a home exercise program. Final analysis was performed for 33 patients, average age 55.8 (range 50–62) years and average follow-up of 35 (range 26–49) months. Patients were followed up at 3, 6, and 12 months and yearly thereafter using the Lysholm Knee Scoring Scale, Tegner Activity Scale (TAS), and visual analog scale (VAS). The analysis was performed using one-way analysis of variance (ANOVA) and Pearson’s correlation coefficient to determine the relationship between Lysholm score and body mass index (BMI).

**Results:**

Patients showed an improvement in Lysholm score, TAS, and VAS, which reached maximum in 6 months and later was accompanied by a decline. However, scores at the final follow-up were significantly better than the pretherapy scores. There was also a progression in arthritis as per Kellgren and Lawrence radiographic classification from median 1 preintervention to median 2 at the final follow-up. A correlation between BMI and Lysholm scores was seen (*r* = 0.47).

**Conclusion:**

Supervised physical therapy with a short course of analgesics followed by a home-based program results in symptomatic and functional improvement over a short-term follow-up; however, osteoarthritis progression continues and is related to BMI.

## Introduction

Degenerative tears of the medial meniscus constitute an important diagnoses in middle-aged patients presenting with knee pain, swelling, and loss of function [1]. Meniscal tears have been known to occur without trauma in physically active individuals as well as in older people and can be recognized as a part of early osteoarthritis [1, 2]. Though the tears can occur in any part of the meniscus, the tear of the medial meniscal root is a relatively newly recognized entity [3]. Root tear has been defined as a radial tear that occurs within 1 cm of the posterior horn insertion [4]. The posterior root attachment of the medial meniscus is, for several reasons, the most likely of all the root attachment sites to undergo failure [5, 6]. The medial meniscus takes more force during weight bearing than the lateral meniscus and is therefore, as a whole, more prone to injury and degeneration [7–9]. The body and posterior horn of the medial meniscus are inherently the least mobile parts and take most of the force applied to the medial compartment [7–9]. Thus it follows that posterior horn and posterior root injuries as a group are more common and are said to have greater functional significance compared with anterior injuries [10]. The posterior root attachment site of the medial meniscus is critical for maintaining hoop strain, preventing extrusion, and preserving meniscal function [5–7, 9]. A posterior root tear of the medial meniscus causes an increase in joint contact pressure and change in knee-joint kinematics [6]. Clinical diagnosis of chronic or degenerative medial meniscal posterior horn and posterior root injuries is often difficult [3, 5]. Primary surgical repair in this degenerative knee is almost impossible because of already degenerated meniscal tissue and low baseline healing potential [3]. Seo et al. [11] performed second-look arthroscopies in 11 patients who underwent arthroscopic repair of posterior root attachment at average 13.4 months postoperatively, and in none of them did the repair heal. Partial meniscectomy may be helpful in the presence of mechanical symptoms; however, there are no clear long-term benefits [5]. There are reports that quadriceps weaken due to knee pain [12], and there is strong evidence that physical training plays an important role in reducing symptoms and improving muscle strength and physical ability and thereby quality of life in patients with medial meniscus tears [13]. Though surgical repair has not shown relief at second-look arthroscopies, there has been an improvement in patient symptoms [11]. The exact reason for this improvement is not known, and hence the question of whether surgery is simply a placebo. Literature regarding nonoperative management of these tears is rather scarce. We therefore decided to investigate the role of physical therapy in managing degenerative posterior root tears of the medial meniscus. The goal of rehabilitation is to regain good knee control, range of motion (ROM), flexibility, and muscle strength and thereby improve knee function [13]. Many authors [1, 13, 14] report the importance of an exercise program tailored for individuals with degenerative meniscal tears to help them attain maximal clinical outcome. Thus, the purpose of this investigation was to evaluate the effect of supervised exercise therapy for nontraumatic radial tears in the medial meniscus root in middle-aged patients. We hypothesized that in degenerative radial tears of the medial meniscus posterior horn, nonoperative treatment with stretching and strengthening exercises and short-term analgesic therapy can effectively reduce pain and improve knee function. However, patients with increased body mass index (BMI) would have an inferior outcome at follow-up.

## Materials and methods

This is a prospective study of 37 consecutive patients who presented to us between January 2005 and May 2007 with symptoms secondary to isolated tears in the medial meniscus posterior horn. The study was performed at the All India Institute of Medical Sciences, New Delhi, India. Institutional ethical committee clearance was obtained, and the study was performed in accordance with the ethical standards of the 1964 Declaration of Helsinki as revised in 2000. Patients were recruited with prior informed consent by the authors. Inclusion criteria were:

(a) medial knee pain without a trauma—daily or almost daily pain experienced during the last 1–3 months

(b) patients with degenerative tear in the posterior root of the medial meniscus as diagnosed by the same musculoskeletal radiologist on magnetic resonance imaging (MRI) (Siemens Symphony 1.5 Tesla Magnetom, Siemens Medical Systems, Erlangen, Germany) with a dedicated transmit/receive extremity coil

(c) knee osteoarthritis grade 0, 1, or 2 on weight-bearing radiographs according to Kellgren and Lawrence (K–L) radiographic classification [15]

(d) patients who consented to follow our protocol of exercise management

(e) minimum follow-up of 2 years.

Exclusion criteria were:

(a) clinical instability around the knee

(b) any other meniscal or ligament pathology on MRI

(c) history of significant trauma, i.e., accidents or serious injuries

(d) patients with varus malalignment with >30 % medial tibial condyle deviation of the mechanical axis and/or varus thrust on gait examination

(d) patellofemoral osteoarthritis on skyline view

(e) history of chronic illness, systemic joint disorders, and collagen disorders

(f) bony lesions around the knee joint

(g) history of previous surgery or arthroscopy around the knee joint

(h) contraindication to physical training.

A thorough history of the nature of pain was elicited from the patient, and a detailed clinical examination was performed. BMI was determined by weight in kilograms divided by the patient’s height in millimeters squared (BMI = kg/m^2^) [5, 16, 17]. BMI was classified according to previously described criteria [5, 15, 16]. Categories were underweight (<18.5); normal weight (18.5–24.9); overweight (25–29.9); obese (30–39.9); morbidly obese (≥40). All patients were diagnosed by the same musculoskeletal radiologist. Full-length standing hip–knee–ankle, anteroposterior (AP), lateral, and skyline radiographs were used to evaluate the mechanical axis and arthritic changes. The severity of arthritic changes was classified according to K–L radiographic classification [15]. A degenerative tear of the medial meniscus root was diagnosed when coronal MRI showed abrupt truncation of the contour of the inner portion of the medial meniscus posterior horn, with loss of normal low-signal-intensity meniscal root as it approached the posterior cruciate ligament (PCL) [9]. Sagittal images showed loss of visualization of the meniscal root anterior to the tibial attachment of the PCL [9]. Assessment for possible medial meniscal extrusion was carried out on the center of the coronal slices, which were seen to pass through the medial collateral ligament [7, 9]. A vertical line was then drawn along the peripheral margin of the medial tibial plateau at the point of transition from horizontal to vertical. The distance between this line and another vertical line drawn along outer margin of the meniscus was then measured. If this distance was ≥3 mm, meniscal extrusion was deemed present [7, 9]. Preintervention MRI in all patients revealed that 24 patients (72.72 %) had concomitant degeneration of the medial meniscus posterior horn, and 18 (54.5 %) had medial extrusion of the medial meniscus >3 mm.

Nonoperative treatments included supervised sessions of physical therapy (Table 1) with scope for individual adaptation thrice a week during the first 6 weeks and then at least twice a week for a further 6 weeks under supervision of a musculoskeletal physiotherapist. Daily analgesics were administered for those 6 weeks and then as required during the follow-up. Celecoxib 200 mg was used unless contraindicated, in which case ibuprofen sustained release 1,600 mg once daily after dinner was the preferred drugs. In case of contraindications to nonsteroidal anti-inflammatory drugs (NSAIDs), paracetamol 4 g/day in divided doses supplemented with tramadol sustained release 100 mg at night was used. The goal of the exercise program was to reduce pain, restore full ROM, and improve knee function. Patients were informed to perform exercises with some strain but almost pain free and without having any negative influence in the affected knee the following day. If the patient was found to tolerate the exercises without any problems, they were instructed to perform the exercises with increasing weights and higher resistance. A written home program was also carried out on other days which comprised strengthening and stretching exercises with 3–10 repetitions once a day. After 12 weeks of supervised therapy, patients were encouraged to continue stretching and strengthening exercises at home (all exercises listed in Table 2, except cycling) once a day for 5 days a week. Patient compliance for home exercises was encouraged and ensured by regular telephone follow-up once a month by the team involved in study. By the end of the study period, four patients were excluded due to noncompliance to inclusion criteria.

**Table 1:** Supervised exercise schedule

Time (weeks)	Exercise	Repetitions
0–12	Stretching of knee extensors and flexors	30 s/muscle group × 3
0–12	Range of movement in hip, knee, and ankle in all directions	30 s/joint × 3 repetitions
0–12	Stationary bicycling	10–20 min gradual increase
0–4	Knee flexion concentrically with two legs and eccentrically with one leg	3 × 10 repetitions
5–12	Knee flexion with one leg using gradually increasing resistance with Thera-Band	3 × 10 repetitions
0–4	Knee extension concentrically with two legs and eccentrically with one leg	3 × 10 repetitions
5–12	Knee extension with one leg using gradually increasing resistance with Thera-Band	3 × 10 repetitions
0–2	Straight leg raises with one leg	3 × 10 repetitions
3–12	Straight leg raises with one leg with weights attached (increase as tolerated)	3 × 10 repetitions
0–4	Mini squats with less than 80° flexion without weights	3 × 10 repetitions
5–12	Mini squats with less than 80° flexion with weights	3 × 10 repetitions

**Table 2:** Total patients classified according to body mass index (BMI)

BMI	Male	Female
Underweight <18.5	1	0
Normal weight 18.5–24.9	5	4
Overweight 25–29.9	3	9
Obese 30–39.9	3	6
Morbidly obese >40	0	2

Before intervention and at 3, 6, and 12 months and final follow-up, all patients were subjected to anteroposterior (AP) and lateral radiographs of the knee joints, visual analog scale (VAS) pain (between 0 and 10 on a 100-mm scale, with 0 being no pain and 10 the most painful), the Lysholm Knee Scoring Scale [18], and Tegner Activity Scale (TAS) [19]. The Lysholm score is a well-validated functional score designed for knee ligament injuries but has also been validated for other knee injuries. The questionnaire considers activities of daily living (ADL) that are important to the patient—namely, limp, support, stair climbing, squatting, locking, instability, pain, and swelling—and are given scores according to the level of function, with maximum being 100 [18]. The Lysholm score has been designed to evaluate knee-ligament injuries and is reported to be sensitive for evaluating patients with meniscal tears [20]. Tegner and Lysholm [19] showed that activity grading is a valuable complement to functional scoring of the knee and designed a scale in which work and sport activities were graded numerically, with zero representing sick leave or disability pension because of knee problems and ten representing competitive sports, soccer, football, rugby (national elite). Knee-joint motion was measured using a manual goniometer. The questionnaires were administered and collected by the authors. Statistical analysis was conducted with an ANOVA test to compare clinical results over time, and Pearson correlation was used to measure the association between BMI and Lysholm score and radiological grading with the use of SPSS software package (version 11.0; SPSS, Chicago, IL, USA). *P* value <0.05 at a 95 % confidence interval (CI) was considered significant.

## Results

During the study period, 37 patients satisfied the study criteria, of whom two patients were not compliant with the exercise schedule and two others were lost to follow-up before 2 years; hence, the total cohort comprised 33 patients for the final study analysis. Of these, 12 were men and 21 were women, with an average age of 55.8 (range 50–62) years. We had an average follow-up of 35 (range 26–49) months. All patients were tolerant to celecoxib or ibuprofen as per the protocol. The patient distribution according to BMI is shown in Table 2. Lysholm score (average 56) at preintervention improved to an average 84 at 6 months, which was a significant improvement (*P* = 0.0001); however, it decreased with time (Table 3) but significantly (*P* = 0.39). Despite the decrease over time, scores at final follow-up were better than the preintervention scores and were consistent with significant improvement. When the Lysholm scores were compared at different time intervals with BMI (Table 4), it followed the same overall trend, with the best scores at 6 months and a subsequent decrease over time. Spearman correlation coefficient showed a moderate correlation of BMI with Lysholm score (*r* = 0.47). Two female patients with a BMI of 38.6 and 41.5 showed a rapid decrease in Lysholm score and radiology grade and underwent total knee arthroplasty (TKA) at 35 and 28 months’ follow-up, respectively.

**Table 3:** Comparison of Lysholm Knee Scoring Scale, Tegner Activity Scale (TAS), and visual analog scale (VAS) over time reported as average (for Lysholm and VAS) and median (for Tegner), with range in parentheses

Time	Lysholm Knee Scoring Scale	Tegner activity scale	VAS (rest)	VAS (activity)
Preintervention	56 (32–73) SD ± 8	2 (0–3)	2 (0–3)	5 (2–7)
3 months	81 (70–94) SD ± 7	3 (2–4)	0 (0–1)	2 (0–2)
6 months	84 (70–95) SD ± 6	4 (4–5)	0 (0–1)	0 (0–2)
12 months	85 (64–93) SD ± 5	4 (3–5)	0 (0–3)	1 (0–3)
Final follow-up	79 (40–91) SD ± 7	4 (1–5)	0 (0–4)	1 (0–7)
*P* value between preintervention and final follow-up	0.0212	0.033	0.0284	0.0397

VAS scores approximated to nearest integer whole number

*SD* standard deviation

**Table 4:** Comparison of body mass index (BMI) with Lysholm Knee Scoring Scale reported as average scores over time

BMI	Preintervention	6 months	12 months	Final follow-up
Underweight <18.5	72	93	92	91
Normal weight 18.5–24.9	66 (62–73) SD ± 2	92 (85–95) SD ± 2	89 (81–93) SD ± 2	85 (76–92) SD ± 4
Overweight 25–29.9	64 (59–71) SD ± 4	90 (81–95) SD ± 3	85 (77–92) SD ± 4	81 (70–90) SD ± 6
Obese 30–39.9	56 (35–68) SD ± 6	83 (78–91) SD ± 3	81 (67–89) SD ± 5	77 (60–87) SD ± 6
Morbidly obese >40	36 (32–40)	76 (74–78)	59 (52–67)	51 (40–62)

*SD* standard deviation

TAS (Table 3), which was median 2 preintervention and corresponded to light work and walking on uneven grounds, improved to median 4 at the latest follow-up, when moderately heavy labor was possible. This improvement was significant when compared over time (*P* = 0.0015). Pain was of acute onset in 19 patients. Pain intensity and frequency were closely related to specific activities such as stair climbing, squatting, and rising from a low chair, all of which require a relatively greater degree of knee flexion and strength. Pain severity and frequency decreased with time (Table 3). VAS, which was mean 2 at rest, became mean 0 at final follow-up; VAS on activity improved from mean 5 to mean 1 at final follow-up. Two patients who underwent TKA showed initial improvement in VAS scores; however, the scores increased with time to reach 7 when they underwent TKA. Patient distribution as graded with the K–L system is shown in Table 5. Median preoperative K–L radiographic score, which was 1 (range 0–2) points, increased to 2 (range 0–4) points at last follow-up and showed significant worsening (*P* = 0.0021). When the Lysholm score was compared between the two groups (meniscal extrusion >3 mm and <3 mm), there was no significant difference (*P* = 0.25) at the latest follow-up. However, the two patients who underwent TKA had an extruded meniscus of 4 and 4.5 mm, respectively.

**Table 5:** Kellgren and Lawrence radiographic classification at initial and final follow-up

Time	K–L 0	K–L 1	K–L 2	K–L 3	K–L 4
Preintervention	8	16	9	0	0
Final follow-up	2	10	12	7	2

## Discussion

It has been shown in cadaver models that tears in the medial meniscus posterior root causes increased contact pressures and kinematic derangements. However, meniscal repairs of these tears may restore the pressure to normal. Though tear repair may be successful in young patients, it may not be possible in a degenerated knee due to low healing potential wherein meniscal degeneration has already set in [4, 5]. Hence, partial meniscectomy has been proposed in this subset of patients to relieve mechanical symptoms [3–5]. Partial meniscectomy provides partial symptomatic relief in most cases, though the arthritic pain persists and does not arrest progression of radiographic osteoarthritis [3–5]. In our study, supervised physical therapy followed by a home-based program resulted in symptomatic and functional improvement over a short-term follow-up. Exercise as a treatment option after meniscal posterior root tears has not been sufficiently studied. Resistance training has been effective in rehabilitation of orthopedic injuries [14]. Aichroth recommends treating meniscal tears detected on MRI with mobility and strengthening exercises and gradually increased resistance training [1]. In a comparative study of operative and nonoperative treatment groups with degenerative medial meniscus tears, Rimington et al. [21] found no significant difference in mean initial and final standardized ADL for knee scores. The mean final Modified Lysholm Knee Scoring Scale (MLKS) in the operative group was significantly greater than in the nonoperative group. In a comparative study by Herlin et al. [13], patients with degenerative medial meniscus tears were randomized to arthroscopic partial meniscectomy or nonoperative exercise program and followed prospectively. At the 6-month follow-up, both groups improved significantly from baseline in Lysholm, VAS, and TAS; however no significant difference was seen between the two groups. MRI was the only imaging modality to confirm the diagnosis in our patients who were to undergo exercise therapy, and in a symptomatic patient when looking for MRI signs. Further, in the hands of a musculoskeletal radiologist, MRI sensitivity for diagnosing medial meniscal root radial tears was around 90 % [22]. 

A root tear is reportedly difficult to diagnose because meniscal tissue is noted only on one side of the tear [23] and may not be visible in about one third of MRI scans [5]. The diagnosis is easier to make medially because of the close anatomic relationship between the posterior horn of the meniscus and the tibial attachment of the PCL [24]. Normally, on 3-mm sagittal images, the meniscus should be seen on the image medial to the PCL attachment; otherwise, a root tear is suspected, which coronal images can confirm [23, 24]. In contrast to sequences for diagnosing meniscal tears with routine knee MRI wherein the sagittal T1-weighted and intermediate images are thought to be the most accurate, interpretation with the coronal T2-weighted sequence in the best for medial meniscal root radial tears. During the course of our study, our team and the musculoskeletal radiologist constantly kept updating the literature in order to improve our diagnostic yield, and we feel that when there is clinical suspicion, 3-mm MRI cuts improve the diagnostic yield. BMI ranges are based on the effect of body weight on disease and death [16]. Osteoarthritis is a common condition related to being overweight and obese [16]. A meniscal lesion has not only been associated with the presence of a degenerative knee, but BMI has also been shown as an independent risk factor for the development of meniscal tears [5, 25]. Menisci are believed to transmit more than half the load to the knee joint [25]. Thus, there is a biomechanical relationship between BMI and meniscal tears [25]. As BMI increases, the strain and torque in the knee joint during rotation likely increases, in theory leading to a higher risk of meniscal injuries [25]. BMI showed moderate correlation with Lysholm scores, and the median score between individuals with a BMI < 24.9 at all times during follow-up were significantly better than for patients with BMI > 24.9. In the study by Ozkoc et al. [5], there was no significant correlation between BMI and radiography and BMI and functional results. However, in their study of the 67 patients, only one had a BMI < 24.9, whereas in our study, ten of 33 patients had a BMI < 24.9. Thus, we agree with previous studies [25] that BMI is closely related to meniscal tears and suggest that a posterior root tear may cause a decreased benefit of exercise in obese patients, increased progression of osteoarthritis, and necessitate a total joint replacement, as seen with two of our patients. Meniscal root attachments present one of the primary factors in maintaining resistance to hoop strain during load bearing. Large radial tears oriented in a perpendicular direction to the circumferential collagen fibrils in the main portion of the meniscus and completely disrupt the ability to resist hoop strain thus often result in major meniscal extrusion [7]. Tears involving the meniscal root (central attachment) are also significantly related to the severity of meniscal extrusion, which is seen in 3 % of patients with minor extrusion and 42 % with major extrusion [7]. Meniscal extrusion, occurring as part of the disease, contributes significantly to radiographic narrowing in patients with osteoarthritis, particularly early in the disease [8]. 

In this study, MRI showed a meniscal extrusion in all patients with K–L 2, and in nine patients with K–L 1; thinning of articular cartilage was observed in no cases. However, at final follow-up, there was no significant difference in Lysholm scores between patients with or without meniscal extrusion. We feel that this may be because as the meniscus is extruded, its periphery, which is supplied with pain fibers, may not be getting compressed between the femoral condyles. Few studies in the literature have dealt with the role of partial meniscectomy after medial meniscus posterior root tear. Ozkoc et al. [5], in a retrospective study, showed that Lysholm scores improved from 53 preoperatively to 67 at an average follow-up of 56 months. However, they also reported that the mean preoperative K–L radiograph grade of 2 (range 0–3) points increased to 3 (range 2–4) points at the latest follow-up, implying continuation of the degenerative process. Han et al. [4], in a retrospective evaluation of 46 cases after partial meniscectomy and at a mean follow-up of 78 months, document that the mean modified Lysholm score significantly increased from a preoperative value of 72 (range 62–78) to a final follow-up value of 77 (range 70–98); 56 % of patients had improvement in pain, 67 % were satisfied with the outcome of the procedure, and 35 % showed radiographic progression of osteoarthritis at a mean follow-up of 77 months. We feel that a radial tear disturbs the hoop stresses important in meniscus function. Also, in partial meniscectomy, additional meniscal tissue is removed, and there is no way restore the hoop stresses important for meniscal function. Conversely, this may lead to progression of osteoarthritis. Kan et al. [26], in a cadaveric study, showed that patients with a radial tear of the medial meniscus posterior horn exhibited significantly greater severity of cartilage degeneration than patients with other types of tears (*P* < 0.05). It has been shown that strength deficits, particularly in the quadriceps, are seen in patients with osteoarthritis [27]. Muscle-strength deficits or dysfunctions may contribute to changes in shock absorption and impact forces, which may result in onset and progress of osteoarthritis [28, 29]. A weak or dysfunctional muscle may be less efficient in reducing impact forces that cross the joint, thus leading to changes in the distribution of stress in the articular cartilage and contributing to subsequent degenerative changes within the joint [29]. Muscle is an adaptable tissue [28]; therefore, exercise training should be considered for patients with medial meniscus posterior horn radial tears to help prevent or delay muscle dysfunction and joint damage. The Lysholm score in our study showed a significant improvement after 3 months of exercise, and this improvement continued with regular home exercises until 6 months. However, after that, the scores showed a slight decrease at 1 year and at final follow-up, but this was not statistically significant. VAS and TAS also showed improvement and followed a pattern similar to Lysholm scores. Average patient age in our study was 55.8 years, and none of them were involved in competitive sports. We had 24 patients with K–L grade 0 or 1 at the beginning of the study, whereas at the last follow-up, it was 12, implying a continuation of the degenerative process. 

There were some limitations to our study: sample size was small, and average follow-up was not long enough. As we were unsure about root-tear repair in a degenerated meniscus and the effectiveness of partial meniscectomy, which we feel has no role in a root tear, we did not have a group for comparison with surgical aspects of management. Neither did we have a group in which patients did not receive treatment, as we felt all patients deserved treatment because there is evidence that exercise improves quadriceps function and, in the long run, knee function. Hence, the most effective measure for delaying the progress of osteoarthritis cannot be commented upon. This may further require a randomized controlled comparative study with long-term follow-up. MRI was used to establish the diagnosis, and there is always the possibility of over- or underdiagnosis [5, 8, 9]. We did not measure the effect of NSAIDs in view of the fact that the patients might develop a placebo effect of pain relief. Patient adherence to an exercise program may be higher in a trial setting than in a routine clinical setting.

Medial meniscus posterior root radial tears are strongly associated with older age and increased BMI. Supervised physical therapy with a short course of analgesics, followed by a home-based program, results in symptomatic and functional improvement over a short-term follow-up; however, progression of osteoarthritis continues and is related to BMI.
